# Lipid indices as simple and clinically useful surrogate markers for insulin resistance in the U.S. population

**DOI:** 10.1038/s41598-021-82053-2

**Published:** 2021-01-27

**Authors:** Juncheol Lee, Bongyoung Kim, Wonhee Kim, Chiwon Ahn, Hyun Young Choi, Jae Guk Kim, Jihoon Kim, Hyungoo Shin, Jun Goo Kang, Shinje Moon

**Affiliations:** 1 Department of Emergency Medicine, Armed Forces Capital Hospital, Seongnam, Republic of Korea; 2 Department of Internal Medicine, Hanyang University College of Medicine, Seoul, Republic of Korea; 3 Department of Emergency Medicine, Hallym University, Chuncheon, Republic of Korea; 4 Department of Emergency Medicine, College of Medicine, Chung-Ang University, Seoul, Republic of Korea; 5 Department of Thoracic and Cardiovascular Surgery, Hallym University College of Medicine, Chuncheon, Republic of Korea; 6 Department of Emergency Medicine, College of Medicine, Hanyang University Guri Hospital, Guri, Republic of Korea; 7 Department of Internal Medicine, Hallym University, Chuncheon, Republic of Korea; 8 Division of Endocrinology and Metabolism, Hallym University College of Medicine, 1, Hallymdaehak-gil, Chuncheon-si, Gangwon-do 24252 Republic of Korea

**Keywords:** Endocrinology, Endocrine system and metabolic diseases, Epidemiology

## Abstract

This study aimed to compare the accuracy of novel lipid indices, including the visceral adiposity index (VAI), lipid accumulation product (LAP), triglycerides and glucose (TyG) index, TyG-body mass index (TyG-BMI), and TyG-waist circumference (TyG-WC), in identifying insulin resistance and establish valid cutoff values. This cross-sectional study used the data of 11,378 adults, derived from the United States National Health and Nutrition Examination Survey (1999–2016). Insulin resistance was defined as a homeostasis model assessment-insulin resistance value above the 75th percentile for each sex and race/ethnicities. The area under the curves (AUCs) were as follows: VAI, 0.735; LAP, 0.796; TyG index, 0.723; TyG-BMI, 0.823, and; TyG-WC, 0.822. The AUCs for TyG-BMI and TyG-WC were significantly higher than those for VAI, LAP, and TyG index (vs. TyG-BMI, p < 0.001; vs. TyG-WC, p < 0.001). The cutoff values were as follows: VAI: men 1.65, women 1.65; LAP: men 42.5, women 42.5; TyG index: men 4.665, women 4.575; TyG-BMI: men 135.5, women 135.5; and TyG-WC: men 461.5, women 440.5. Given that lipid indices can be easily calculated with routine laboratory tests, these values may be useful markers for insulin resistance risk assessments in clinical settings.

## Introduction

Insulin resistance (IR) is a pathological situation, in which there is a lack of physiological response to insulin acting on peripheral tissues^1,2^. Insulin resistance reduces glucose utilization in the muscles and fats and increases gluconeogenesis in the liver, leading to metabolic and hemodynamic disturbances known as metabolic syndrome, which is a major risk factor for coronary heart disease and cerebrovascular disease^1–7^. Considering the prevalence of insulin resistance and metabolic syndromes, it would be necessary to detect insulin resistance early even in healthy individuals^8^.

Insulin resistance was initially evaluated using the pancreatic suppression test, hyperinsulinemic euglycemic clamp technique (HIEG clamp), or minimal model approximation of the metabolism of glucose (MMAMG)^9–11^. However, these methods are invasive, complicated, expensive, and difficult to use clinically^12^. For these reasons, indices that measure insulin resistance indirectly have been developed. The homeostasis model for IR (HOMA-IR), which uses fasting blood glucose levels and insulin concentration as variables, was developed in 1985 and has been widely used to estimate IR^13^. However, a significant drawback of HOMA-IR is the lack of a standard assay for the measurement of fasting insulin concentration^14^. Therefore, considering these concerns regarding standardization, the HOMA-IR has a significant limitation in establishing an overall acceptable reference value. Furthermore, while several studies have defined IR as a value greater than the 75th percentile value of the HOMA-IR in individuals without diabetes mellitus, the reported cutoff values vary widely, ranging from 2.0 to 3.8^12,15–19^. Given that the measurement of fasting insulin concentration is cumbersome and expensive, the HOMA-IR is not routinely measured in the clinical setting^20^.

Therefore, insulin-free equations for estimating IR, such as lipid indices, were developed. Lipid indices include visceral adiposity index (VAI), lipid accumulation product (LAP), and triglycerides and glucose (TyG) index^21–25^. These parameters were proposed as a useful surrogate measure of insulin resistance^25–28^. In addition, several studies have evaluated modified indices that combine TyG index and obesity indices such as body mass index (BMI) and waist circumference (WC)^29–31^. However, limited evidence is available regarding the discriminatory accuracy and cutoff values of these novel lipid indices for detecting insulin resistance.

Therefore, this study aimed to compare the accuracy of novel lipid indices in identifying insulin resistance using a representative sample of the US population and establish valid cutoff values for IR.

## Results

**Figure 1:**
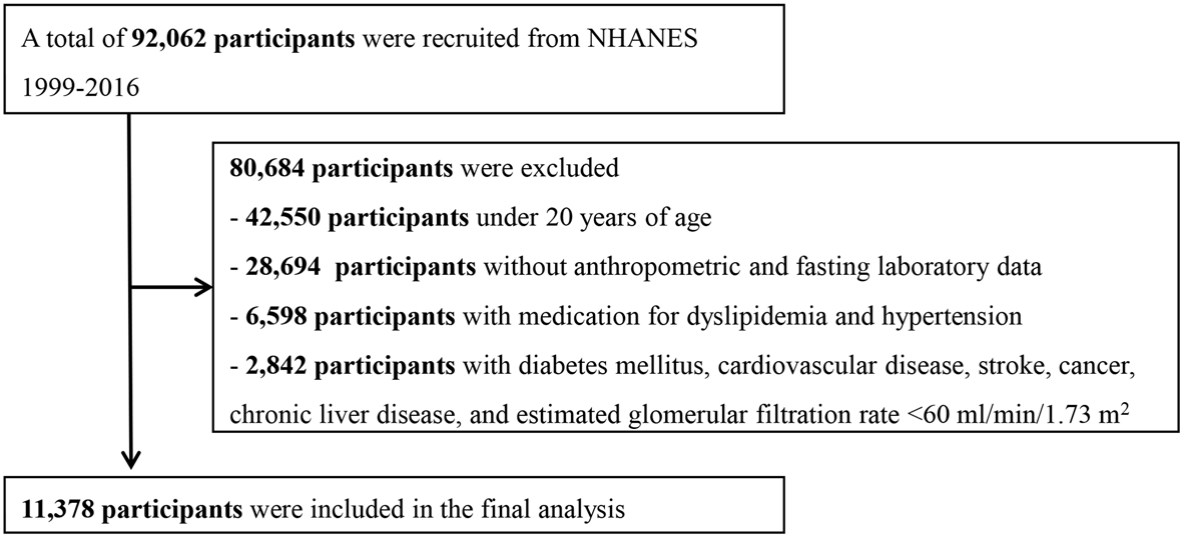
Flowchart showing the final selection process.

The study included 11,378 adults (men 5478, women 7900; mean age, 40 years) from the National Health and Nutrition Examination Survey (NHANES) 1999–2016 (Fig. 1). Participants’ demographic and clinical characteristics were compared based on the presence or absence of IR, and the results are shown in Table 1. Age, BMI, WC, and blood pressure were higher in participants with insulin resistance. In addition, blood tests demonstrated high values of fasting glucose, hemoglobin A1C, fasting insulin, total cholesterol, and triglycerides, and low value of high-density lipoprotein (HDL) cholesterol, in participants with IR. Data (median with interquartile range) of each parameter according to race/ethnicity and sex are summarized in Table 2.

**Table 1:** Baseline characteristics of the participants in NHANES 1999–2016.

Characteristics	Normal (N = 8524)	Insulin resistance* (N = 2854)	p-value
Age	40.0 ± 14.8	39.6 ± 14.3	0.239
Sex (men)	4106 (48.2%)	1372 (48.1%)	0.946
**Race**			0.999
Hispanics	2537 (29.8%)	847 (29.7%)	
Non-Hispanic Whites	3548 (41.6%)	1189 (41.7%)	
Non-Hispanic Blacks	1644 (19.3%)	550 (19.3%)	
Other race^†^	795 (9.3%)	268 (9.4%)	
BMI, kg/m^2^	26.1 ± 4.9	32.6 ± 7.1	< 0.001
WC, cm	90.6 ± 12.4	106.5 ± 15.2	< 0.001
Smoking (≥ 100 cigarettes in life)	3613 (42.4%)	1156 (40.5%)	0.081
Systolic blood pressure, mmHg	116.7 ± 17.3	121.1 ± 17.1	< 0.001
Diastolic blood pressure, mmHg	68.6 ± 12.0	71.8 ± 12.8	< 0.001
Fasting glucose, mg/dL	93.8 ± 9.2	100.9 ± 9.7	< 0.001
Fasting insulin, μU/mL	7.5 ± 3.3	23.0 ± 13.6	< 0.001
HbA1c, %	5.3 ± 0.4	5.4 ± 0.4	< 0.001
Total cholesterol, mg/dL	195.4 ± 41.3	200.3 ± 40.7	< 0.001
Triglycerides, mg/dL	112.5 ± 89.7	164.0 ± 115.0	< 0.001
HDL-cholesterol, mg/dL	56.9 ± 16.2	47.4 ± 13.2	< 0.001
HOMA-IR	1.6 ± 0.7	5.5 ± 3.5	< 0.001
VAI	1.6 ± 2.0	2.9 ± 2.7	< 0.001
LAP	39.4 ± 41.5	83.5 ± 61.2	< 0.001
TyG index	4.5 ± 0.3	4.8 ± 0.3	< 0.001
TyG-BMI	119.0 ± 24.8	155.4 ± 34.3	< 0.001
TyG-WC	413.0 ± 68.5	509.0 ± 79.5	< 0.001

Data are presented as mean ± standard deviation or number (%).

*BMI* body mass index, *WC* waist circumference, *BP* blood pressure, *HbA1c* hemoglobin A1c, *HDL* high-density lipoprotein; *HOMA-IR* homeostasis model assessment-insulin resistance, *VAI* visceral adiposity index, *LAP* lipid accumulation product, *TyG index* triglycerides and glucose index.

*Insulin resistance was defined as a HOMA-IR value above the 75th percentile for each sex and race/ethnicity.

^†^Other race included non-Hispanic Asian and multi-racial Americans.

**Table 2:** Distribution of indirect parameters for insulin resistance according to race/ethnicity and sex.

Indirect parameters for insulin resistance	Total	Hispanic	Non-Hispanic white	Non-Hispanic black	Other race*
	Median (IQR)	Median (IQR)	Median (IQR)	Median (IQR)	Median (IQR)
**HOMA-IR**					
Men	1.98 (1.27–3.24)	2.34 (1.49–3.62)	1.86 (1.20–3.07)	1.82 (1.14–3.18)	1.94 (1.18–2.96)
Women	1.87 (1.21–3.06)	2.24(1.46–3.44)	1.60 (1.05–2.53)	2.17 (1.40–3.59)	1.59 (1.05–2.53)
**VAI**					
Men	1.4 (0.8–2.3)	1.6 (1.0–2.7)	1.4 (0.9–2.5)	1.0 (0.6–1.5)	1.3 (0.8–2.3)
Women	1.4 (0.9–2.2)	1.7 (1.0–2.6)	1.4 (0.9–2.3)	1.0 (0.7–1.7)	1.2 (0.8–2.1)
**LAP**					
Men	38 (20–66)	45 (26–72)	41 (23–72)	26 (14–47)	30 (14–56)
Women	35 (20–63)	43 (24–71)	34 (19–63)	31 (18–52)	25 (14–47)
**TyG index**					
Men	4.64 (4.45–4.85)	4.70 (4.52–4.90)	4.65 (4.47–4.85)	4.52 (4.34–4.7)	4.64 (4.46–4.87)
Women	4.535 (4.35–4.74)	4.62 (4.42–4.81)	4.55 (4.37–4.75)	4.41 (4.26–4.59)	4.50 (4.33–4.72)
**TyG-BMI**					
Men	125 (108–144)	131 (114–147)	125 (108–144)	120 (104–143)	115 (100–133)
Women	122 (103–147)	129 (111–151)	118 (100–143)	130 (108–155)	106 (91–127)
**TyG-WC**					
Men	446 (391–499)	456 (409–503)	455 (400–510)	416 (364–483)	416 (365–466)
Women	415 (362–477)	431 (381–488)	408 (358–473)	421 (365–483)	374 (332–436)

*IQR* interquartile range, *HOMA-IR* homeostasis model assessment-insulin resistance, *VAI* visceral adiposity index, *LAP* lipid accumulation product, *TyG index* triglycerides and glucose index, *BMI* body mass index, *WC* waist circumference.

*Other race included non-Hispanic Asian and multi-racial Americans.

**Figure 2:**
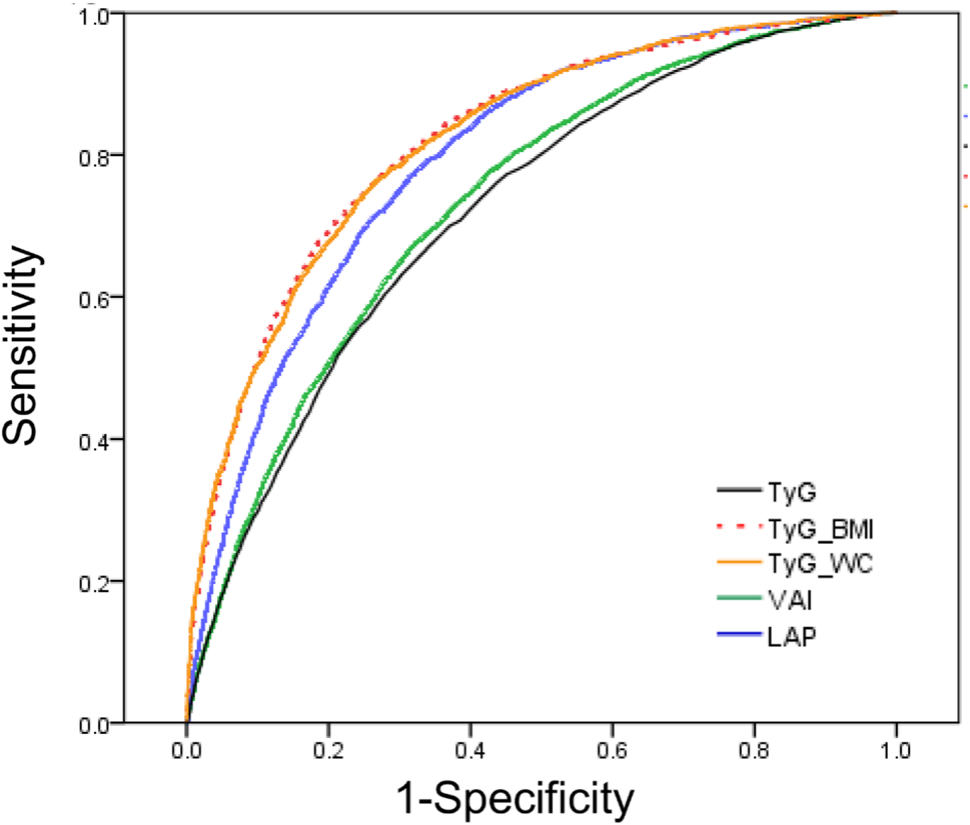
Receiver operating characteristic (ROC) curves of lipid indices for insulin resistance.

The receiver operating characteristic (ROC) curve for IR is presented in Fig. 2. The AUC was 0.723 for TyG index and 0.735 for VAI (Table 3). The AUC of LAP (0.796) was significantly higher than that of TyG index (p < 0.001). However, the AUCs of TyG-BMI (0.823) and TyG-WC (0.822) were significantly higher than that of LAP (vs. TyG-BMI, p < 0.001; vs. TyG-WC, p < 0.001). Subgroup analysis according to sex and race/ethnicities showed that TyG-BMI and TyG-WC had the highest AUC in every subgroup. Further analysis using 1:1 propensity score matching (PSM) data with age, sex, and race/ethnicities showed similar results (Table 3). The cutoff values of each lipid index were as follows: VAI: men 1.65, women 1.65; LAP: men 42.5, women 42.5; TyG index: men 4.665, women 4.575; TyG-BMI: men 135.5, women 135.5; and TyG-WC: men 461.5, women 440.5. The cutoff values with their corresponding sensitivity, specificity, and odds ratio (OR) of insulin resistance according to sex and race/ethnicities are summarized in Table 4.

**Table 3:** Area under the curve for each parameter for insulin resistance according to race/ethnicity and sex.

	VAI	LAP	TyG index	TyG_BMI	TyG_WC
	AUC (95% CI)	AUC (95% CI)	AUC (95% CI)	AUC (95% CI)	AUC (95% CI)
**Total**	0.735 (0.725–0.746)	0.796 (0.787–0.805)	0.723 (0.712–0.733)	0.823 (0.814–0.832)	0.822 (0.813–0.831)
Men	0.735 (0.720–0.750)	0.800 (0.787–0.813)	0.727 (0.712–0.742)	0.829 (0.816–0.841)	0.828 (0.816–0.841)
Women	0.735 (0.721–0.750)	0.793 (0.780–0.806)	0.726 (0.711–0.741)	0.819 (0.807–0.832)	0.825 (0.813–0.837)
**Hispanics**					
Men	0.733 (0.706–0.760)	0.788 (0.764–0.812)	0.730 (0.702–0.757)	0.824 (0.801–0.847)	0.818 (0.795–0.841)
Women	0.718 (0.691–0.745)	0.773 (0.749–0.797)	0.711 (0.684–0.738)	0.818 (0.796–0.840)	0.814 (0.792–0.837)
**Non-Hispanic Whites**					
Men	0.754 (0.731–0.776)	0.82 (0.801–0.839)	0.742 (0.720–0.765)	0.847 (0.829–0.865)	0.852 (0.833–0.870)
Women	0.759 (0.737–0.780)	0.814 (0.795–0.833)	0.753 (0.731–0.774)	0.840 (0.822–0.858)	0.842 (0.824–0.860)
**Non-Hispanic Blacks**					
Men	0.733 (0.698–0.768)	0.815 (0.785–0.845)	0.730 (0.696–0.765)	0.823 (0.793–0.853)	0.829 (0.801–0.858)
Women	0.722 (0.689–0.755)	0.778 (0.748–0.809)	0.710 (0.676–0.744)	0.795 (0.765–0.825)	0.810 (0.781–0.839)
Other race*					
Men	0.734 (0.687–0.781)	0.794 (0.750–0.837)	0.712 (0.663–0.761)	0.808 (0.761–0.855)	0.830 (0.787–0.872)
Women	0.773 (0.729–0.818)	0.824 (0.783–0.864)	0.758 (0.711–0.805)	0.846 (0.808–0.885)	0.847 (0.808–0.885)
**PSM data**					
Total	0.732 (0.719–0.745)	0.790 (0.778–0.802)	0.716 (0.703–0.729)	0.818 (0.807–0.829)	0.818 (0.807–0.828)
Men	0.726 (0.707–0.745)	0.789 (0.772–0.805)	0.718 (0.698–0.737)	0.819 (0.803–0.835)	0.818 (0.802–0.833)
Women	0.737 (0.719–0.755)	0.792 (0.776–0.809)	0.722 (0.704–0.740)	0.817 (0.802–0.833)	0.827 (0.812–0.842)

*HOMA-IR* homeostasis model assessment-insulin resistance, *VAI* visceral adiposity index, *LAP* lipid accumulation product, *TyG index* triglycerides and glucose index, *BMI* body mass index, *WC* waist circumference, *AUC* area under the curve, *PSM* propensity score matching with age, sex, and race/ethnicity.

*Other race included non-Hispanic Asian and multi-racial Americans.

**Table 4:** Cutoff values for each parameter and their corresponding sensitivity, specificity, and odds ratios for insulin resistance.

Indirect parameters for insulin resistance	Men		Women	
	Cutoff value (sensitivity, Specificity, %)	Odds ratio* (95% CI)	Cutoff value (sensitivity, Specificity, %)	Odds ratio* (95% CI)
**VAI**	1.65 (67.3, 69.0)	4.71 (4.08–5.44)	1.65 (66.0, 68.2)	4.27 (3.71–4.90)
Hispanics	1.65 (76.0, 62.0)	5.22 (3.98–6.84)	1.95 (64.0, 66.5)	3.51 (2.75–4.48)
Non-Hispanic Whites	1.65 (72.1, 66.9)	4.76 (3.81–5.94)	1.55 (73.0, 66.2)	4.98 (4.01–6.19)
Non-Hispanic Blacks	1.25 (62.5, 73.3)	4.61 (3.32–6.40)	1.15 (69.6, 64.3)	4.09 (2.96–5.65)
Other race^†^	1.35 (73.8, 61.1)	4.54 (2.84–7.25)	1.35 (77.9, 65.8)	6.04 (3.72–9.81)
**LAP**	42.5 (80.0, 66.4)	8.31 (7.06–9.78)	42.5 (74.4, 70.1)	6.97 (6.01–8.08)
Hispanics	49.5 (76.3, 66.8)	6.40 (4.86–8.43)	48.5 (75.9, 66.2)	5.99 (4.59–7.83)
Non-Hispanic Whites	42.5 (85.4, 63.4)	9.34 (7.14–12.23)	43.5 (75.2, 73.6)	7.90 (6.31–9.89)
Non-Hispanic Blacks	33.5 (78.3, 74.3)	10.27 (7.11–14.84)	36.5 (73.6, 68.8)	6.48 (4.61–9.12)
Other race^†^	32.5 (82.8, 66.5)	9.94 (5.84–16.94)	28.5 (86.3, 68.3)	13.06 (7.40–23.03)
**TyG index**	4.665 (71.9, 61.8)	4.26 (3.68–4.92)	4.575 (69.2, 63.2)	4.10 (3.56–4.72)
Hispanics	4.755 (70.3, 66.8)	4.80 (3.71–6.22)	4.665 (66.3, 64.2)	3.33 (2.60–4.26)
Non-Hispanic Whites	4.665 (76.6, 60.7)	4.51 (3.59–5.68)	4.605 (71.5, 66.6)	4.67 (3.77–5.78)
Non-Hispanic Blacks	4.565 (68.4, 66.2)	4.07 (2.93–5.65)	4.455 (68.6, 64.2)	4.30 (3.12–5.93)
Other race^†^	4.675 (66.7, 61.1)	3.44 (2.21–5.35)	4.565 (71.2, 67.7)	4.83 (3.06–7.63)
**TyG-BMI**	135.5 (72.5, 77.2)	8.62 (7.42–10.00)	135.5 (71.9, 76.9)	9.10 (7.84–10.57)
Hispanics	139.5 (72.7, 77.4)	8.32 (6.36–10.88)	135.5 (78.2, 70.6)	7.85 (5.98–10.31)
Non-Hispanic Whites	137.5 (72.6, 79.9)	9.17 (7.29–11.54)	132.5 (73.9, 79.8)	10.8 (8.64–13.58)
Non-Hispanic Blacks	132.5 (71.5, 77.9)	8.49 (6.04–11.94)	144.5 (68.6, 76.7)	7.18 (5.19–9.93)
Other race^†^	124.5 (72.1, 77.6)	8.52 (5.34–13.59)	111.5 (79.1, 73.5)	10.87 (6.60–17.91)
**TyG-WC**	461.5 (80.4, 70.2)	10.33 (8.74–12.20)	440.5 (76.4, 73.6)	9.38 (8.05–10.94)
Hispanics	477.5 (75.6, 73.8)	9.05 (6.82–12.00)	462.5 (72.2, 76.0)	8.10 (6.22–10.55)
Non-Hispanic Whites	485.5 (76.0, 78.5)	10.59 (8.32–13.47)	440.5 (76.6, 76.6)	10.13 (8.03–12.78)
Non-Hispanic Blacks	456.5 (72.3, 80.0)	9.72 (6.84–13.80)	462.5 (69.6, 78.4)	8.15 (5.83–11.40)
Other race^†^	451.5 (75.4, 80.4)	13.08 (7.88–21.70)	401.5 (77.9, 76.7)	11.69 (6.96–19.62)

*HOMA-IR* homeostasis model assessment-insulin resistance, *VAI* visceral adiposity index, *LAP* lipid accumulation product, *TyG index* triglycerides and glucose index, *BMI* body mass index, *WC* waist circumference.

*Adjusted for Age, race, smoking status, and blood pressure.

^†^Other race included non-Hispanic Asian and multi-racial Americans.

## Discussion

In this study, we investigated the discriminatory accuracy of novel lipid indices for IR and confirmed that LAP showed significantly higher AUC than TyG index and VAI. There was a significant increase in AUC when BMI or WC was combined with TyG index, exhibiting an even higher discriminatory accuracy than that of LAP. Another important aspect of this study is that the cutoff value of each parameter for IR was presented using large-scale data, facilitating the clinical application of each parameter.

Although the mechanism through which lipid indices cause IR remains unclear, numerous studies have reported that glucolipotoxicity is a key mechanism in the modulation of IR^32,33^. Ectopic lipid accumulation in the liver and skeletal muscle tissue activates pathways that are associated with IR, leading to a decrease in the glucose uptake in muscle tissue and glycogen synthesis in the liver^34–37^. Insulin resistance in muscle tissue due to ectopic lipid accumulation increases hepatic lipogenesis and leads to IR in the liver and hyperlipidemia^38–40^. In addition, macrophage infiltration into white adipose tissue increases lipolysis, which stimulates hepatic triglyceride synthesis, thereby, promoting hyperlipidemia^33^. Macrophage-induced lipolysis in white adipose tissue also leads to increased hepatic gluconeogenesis and results in hyperglycemia through increased fatty acid delivery to the liver, which results in increased glycerol conversion to glucose^41–43^.

VAI uses BMI, WC, and triglyceride and HDL cholesterol levels to evaluate IR and was proposed by Amato et al. in 2010^21^. First proposed by Kahn et al. using the NHANES data^22,23^, LAP is calculated using WC and fasting triglyceride levels. In a previous study conducted by Amato et al., VAI showed a significant inverse correlation with insulin sensitivity measured using a HIEG clamp, providing evidence that VAI can be a surrogate marker for IR^21^. In addition, VAI was reported to be associated with the glucose distribution rate evaluated through the HIEG clamp test in a study on patients with type 1 diabetes mellitus (DM)^26^, and it was shown to be inversely correlated with HIEG clamp tested insulin sensitivity in studies conducted on women with polycystic ovary syndrome (PCOS) in South Korea and China^27,44^. In the case of LAP, a small-scaled study with PCOS patients demonstrated a modest inverse correlation with insulin sensitivity measured through HIEG clamp test^27^. However, most of these studies using the HIEG clamp test had a small sample, and the clinical application of VAI and LAP requires an investigation of appropriate cutoff values through large scale population-based studies. In addition to studies measuring insulin resistance directly, there are numerous studies on the accuracy of VAI and LAP in assessing HOMA-IR defined insulin resistance. However, studies on cutoff values were mainly conducted on a small number of PCOS patients^45–47^. Thus, a study with a larger population with healthy adults is necessary. The current study is significant as it presents the cutoff values of VAI and LAP by sex, using a large and healthy population. Interestingly, the VAI cutoff value identified in this study is similar to the VAI cutoff value of 1.6–1.8 reported in the previous studies with PCOS patients^27,45,46^. However, in the case of LAP, previous studies have reported diverse cutoff values ranging from 18.5 to 33.8, and this study shows a higher cutoff value than that reported in the preceding studies^27,45,46^.

TyG index was proposed as a useful surrogate measure of IR by Guerrero-Romero et al. in 2008^24^. Despite the small scale of previous studies, TyG index displayed an inverse correlation with insulin sensitivity measured through HIEG clamp and MMAMG^25,28^. Furthermore, various epidemiological studies have reported that TyG index is associated with the incidences of cardiovascular disease (CVD) and DM, indicating that TyG index is able to predict diseases that result from IR^48–50^. However, there are a few studies on the estimation of the cutoff value of TyG index for IR. Guerrero-Romero et al. suggested that the best value of the TyG index for the diagnosis of IR was 4.68 using the HIEG clamp test with a small sample size^25^. In a study with Korean NHANES, the cutoff values for metabolic syndrome, which is a pathological condition associate with IR, were 4.76 in men and 4.71 in women^19^. In a prospective cohort study with Korean population, the cutoff value to predict DM was 4.69^49^. In our study, the cutoff value of TyG index was 4.66 in men and 4.57 in women, which is similar to those of previous studies.

Recently, there have been studies on indices that combine adiposity status with the TyG index^29–31^. Considering that adipose tissues secrete inflammatory cytokines, adipokines, and reactive oxygen species, contributing to a variety of metabolic problems^51–53^, compound indices with TyG index and obesity parameters such as BMI and WC might be better indicators of IR than TyG index alone. Several studies reported that the compound indices were significantly associated with metabolic abnormalities such as high blood pressure, nonalcoholic fatty liver disease, prediabetes, DM, and hyperuricemia^29–31,54–56^. In addition, recent studies have indicated that TyG-BMI or TyG-WC is more effective in the identification of IR than VAI, LAP, and TyG index^29,31^. Er et al. reported the AUCs for IR were 0.734 for VAI, 0.761 for LAP, 0.708 for TyG index, 0.801 for TyG-BMI, and 0.772 for TyG-WC and proposed TyG-BMI as a clinically useful surrogate marker for the early identification of IR^29^. Lim et al. reported that the AUCs for TyG-BMI and TyG-WC (0.748 and 0.731, respectively) were larger than that for TyG index (0.690)^31^. However, such studies were conducted only in Asian populationss, and no studies have been conducted on other ethnic populations. Therefore, the current study is meaningful as it confirms using large-scale data that TyG-BMI or TyG-WC can be an effective surrogate marker for IR in the US population with various races/ethnicities. Nonetheless, to accurately assess the correlation of IR with TyG-BMI and TyG-WC, verification through the HIEG clamp test is required as has been performed for HOMA-IR and TyG index.

The present study has several strengths. This study is the largest to evaluate the performance of the novel lipid indices to identify insulin resistance in the general US population. In addition, this study conducted various subgroup analyses of IR, with age, sex, and PSM data, to minimize the bias caused by heterogeneity due to demographic characteristics. Moreover, it is important to propose valid cutoff values for each lipid index so that they can be used as a reference in clinical settings for identifying groups at risk for IR. To the best of our knowledge, this is the first study that evaluated the performance and cutoff values of TyG-BMI and TyG-WC in a non-Asian population. However, considering that this is a cross-sectional study, further prospective studies are required to validate the relationship between each surrogate measure and cardiovascular risk factors.

## Conclusion

The present study supports the clinical relevance of novel lipid indices in identifying IR in the general US population. Considering that lipid indices can be easily calculated with routine laboratory tests, they can be useful markers of insulin resistance risk assessments in clinical settings. Moreover, the cutoff values presented in our study may be useful in interpreting the results of lipid indices for IR.

## Methods

### Study population

The NHANES is a cross-sectional study that uses a representative sample of the population living in the United States. The NHANES, administered by the Centers for Disease Control and Prevention every two years, consists of health and nutrition surveys, physical examinations, and laboratory tests. Of the 92,062 individuals who participated in the NHANES between 1999 and 2016, 11,378 adult participants were included in this study after excluding those who were aged < 20 years (n = 42,550), those with missing or incomplete anthropometric and fasting laboratory data (n = 28,694), those on medication for dyslipidemia and hypertension (n = 6598), those with DM, CVD, stroke, cancer, chronic liver disease, and estimated glomerular filtration rate < 60 ml/min/1.73 m^2^ (n = 2842) (Fig. 1).

### Anthropometric and laboratory measurements

Waist circumference was measured using a flexible tape between the uppermost lateral border of the right ilium and that of the left ilium. BMI was defined as the weight in kilograms divided by the height in meters squared (kg/m^2^). Blood pressure was measured 3 times in the sitting position, with at least 5 min of rest in between each reading. The mean value of the three recorded blood pressure readings was used in this study. Fasting blood glucose and lipid levels were measured using the enzymatic method, and fasting insulin was measured using an immune-enzymometric assay. Detailed sample collection and processing instructions are described in the NHANES Laboratory Procedures Manual^57^.

### Calculation of parameters for insulin resistance

Parameters for insulin resistance were calculated as follows^21–25,29,58^:

$${\text{HOMA-IR }} = {\text{ Fasting}}\;{\text{insulin }}\left( \upmu{\text{IU}}/{\text{mL}} \right) \, \times {\text{ Fasting}}\;{\text{glucose }}\left( {\text{mmol}}/{\text{L}} \right)/{22.5},$$

$${\text{VAI }} = \, \left( {{\text{WC }}\left( {{\text{cm}}} \right)/\left( {{39}.{68} + \left( {{1}.{88 } \times {\text{ BMI}}} \right)} \right)} \right) \, \times \, \left( {{\text{triglycerides }}\left( {{\text{mmol}}/{\text{L}}} \right)/{1}.0{3}} \right) \, \times \, \left( {{1}.{31}/{\text{HDL-C }}\left( {{\text{mmol}}/{\text{L}}} \right)} \right){\text{ for men}},{\text{ or }}\left( {{\text{WC in centimeter}}/\left( {{36}.{58} + \left( {{1}.{89 } \times {\text{ BMI}}} \right)} \right)} \right) \, \times \, \left( {{\text{triglycerides }}\left( {{\text{mmol}}/{\text{L}}} \right)/0.{81}} \right) \, \times \, \left( {{1}.{51}/{\text{ HDL-C }}\left( {{\text{mmol}}/{\text{L}}} \right)} \right){\text{ for women}},$$

$${\text{LAP }} = \, \left( {{\text{WC in centimeter}}\, - \,{65}} \right) \, \times \, \left( {{\text{triglycerides }}\left( {{\text{mmol}}/{\text{L}}} \right)} \right){\text{ for men}},{\text{ or }}\left( {{\text{WC }}\left( {{\text{cm}}} \right)\, - \,{58}} \right) \, \times \, \left( {{\text{triglycerides }}\left( {{\text{mmol}}/{\text{L}}} \right)} \right){\text{ for women}},$$

$${\text{TyG index}} = {\text{ Ln }}\left( {{\text{fasting glucose }}\left( {{\text{mg}}/{\text{dL}}} \right) \, \times {\text{ triglycerides }}\left( {{\text{mg}}/{\text{dL}}} \right)} \right)/{2},$$

$${\text{TyG - BMI }} = {\text{ Ln }}\left( {{\text{fasting glucose }}\left( {{\text{mg}}/{\text{dL}}} \right) \, \times {\text{ triglycerides }}\left( {{\text{mg}}/{\text{dL}}} \right)} \right)/{2 } \times {\text{ BMI}},$$

$${\text{TyG - WC }} = {\text{ Ln }}\left( {{\text{fasting glucose }}\left( {{\text{mg}}/{\text{dL}}} \right) \, \times {\text{ triglycerides }}\left( {{\text{mg}}/{\text{dL}}} \right)} \right)/{2 } \times {\text{ WC }}\left( {{\text{cm}}} \right).$$

We define IR as a HOMA-IR value above the 75th percentile for each race/ethnicity and sex (Hispanics: Men > 3.62, Women > 3.44; Non-Hispanic Whites: Men > 3.07, Women > 2.53; Non-Hispanic Blacks: Men > 3.18, Women > 3.59; other race: Men > 2.96, Women > 2.53)^12,15^.

### Statistical analysis

Data were presented as mean with standard deviation, or number with prevalence (%) of IR status. Between groups, the differences were determined using t-tests and a Pearson chi-square test. The values of each lipid index for IR were presented as median and interquartile range. To compare the relative diagnostic strength of each lipid index for insulin resistance, AUC was compared using the ROC curve; de Long’s test was used to identify the surrogate measures that were significantly superior for insulin resistance. The cutoff value of each lipid index was determined as the value with the highest Youden index score. Considering the heterogeneity of demographic characteristics such as sex and race/ethnicities, subgroup analyses for IR were performed. Further analysis was performed by 1:1 PSM with age, sex, and race/ethnicities. This was performed using the “MatchIt” package with nearest-neighbor 1-to-1 matching^59^. Furthermore, OR of HOMA-IR defined IR was checked using the multivariate logistic regression models based on the estimated cutoff values. Statistical analysis was performed using IBM SPSS Statistics ver. 24.0 (IBM Co., Armonk, NY, USA) and R ver. 3.1.0 (R Foundation for Statistical Computing, Vienna, Austria; www.r-project.org). The results were considered statistically significant if the p-value was less than 0.05.

### Ethics statement

This study was approved by the institutional review board of Kangnam Sacred Heart Hospital (IRB No. HKS 2017-07-007) and the NHANES was approved by the Research Ethics Review Board of the National Center for Health Statistics, US Centers for Disease Control and Prevention (NHANES 1999–2004, Protocol #98-12; NHANES 2005–2010, Protocol #2005–06; NHANES 2011–2016, Protocol #2011-17). All participants volunteered and provided written informed consent before enrolment. All participants’ records were anonymized before being accessed by the authors. All methods were carried out in accordance with the principles contained in the Declaration of Helsinki.
